# Modelling Age-Related Changes in the Pharmacokinetics of Risperidone and 9-Hydroxyrisperidone in Different CYP2D6 Phenotypes Using a Physiologically Based Pharmacokinetic Approach

**DOI:** 10.1007/s11095-020-02843-7

**Published:** 2020-05-31

**Authors:** Lisa Alina Kneller, Georg Hempel

**Affiliations:** Institute of Pharmaceutical and Medical Chemistry, Clinical Pharmacy, University of Münster, Corrensstr. 48, 48149 Münster, Germany

**Keywords:** CYP2D6 polymorphism, elderly, PBPK, pharmacokinetics, risperidone

## Abstract

**Purpose:**

Dose-optimization strategies for risperidone are gaining in importance, especially in the elderly. Based on the genetic polymorphism of cytochrome P 450 (CYP) 2D6 genetically and age-related changes cause differences in the pharmacokinetics of risperidone and 9-hydroxyrisperidone. The goal of the study was to develop physiologically based pharmacokinetic (PBPK) models for the elderly aged 65+ years. Additionally, CYP2D6 phenotyping using metabolic ratio were applied and different pharmacokinetic parameter for different age classes predicted.

**Methods:**

Plasma concentrations of risperidone and 9-hydroxyrisperidone were used to phenotype 17 geriatric inpatients treated under naturalistic conditions. For this purpose, PBPK models were developed to examine age-related changes in the pharmacokinetics between CYP2D6 extensive metabolizer, intermediate metabolizer, poor metabolizer, (PM) and ultra-rapid metabolizer.

**Results:**

PBPK-based metabolic ratio was able to predict different CYP2D6 phenotypes during steady-state. One inpatient was identified as a potential PM, showing a metabolic ratio of 3.39. About 88.2% of all predicted plasma concentrations of the inpatients were within the 2-fold error range. Overall, age-related changes of the pharmacokinetics in the elderly were mainly observed in Cmax and AUC. Comparing a population of young adults with the oldest-old, Cmax of risperidone increased with 24–44% and for 9-hydroxyrisperidone with 35–37%.

**Conclusions:**

Metabolic ratio combined with PBPK modelling can provide a powerful tool to identify potential CYP2D6 PM during therapeutic drug monitoring. Based on genetic, anatomical and physiological changes during aging, PBPK models ultimately support decision-making regarding dose-optimization strategies to ensure the best therapy for each patient over the age of 65 years.

## Introduction

The world’s population is aging. In Germany, the number of people aged 65 years and above was reported at 17.8 million (21%) in 2018, exhibiting an upward trend (1,2). Thereby, the age-related comorbidities such as psychotic disorders will rise and the craving for individual treatment in geriatric patients is gaining in importance. One potential treatment option in geriatric patients is risperidone, an atypical antipsychotic. In Norway, 30% of the overall number of risperidone-treated patients are 65 years or older (3). Despite the growing population of elderly individuals, they are often omitted from clinical trials (4). Since psychotropic agents often exhibit great inter-individual variability, only little is known about the impact of aging on the pharmacokinetics (PK) of risperidone and its active metabolite 9-hydroxyrisperidone.

In general, advanced age is characterized by anatomical, physiological, and biochemical changes potentially influencing drug relevant PK. Besides decreased kidney weight (5), reduced renal blood flow (6), reduced glomerular filtration rate (7), reductions in liver volume and blood flow are also affected (8–10). Based on the age-related decline in liver volume, geriatric patients are potentially at a higher risk to adverse drug reactions (ADR) because of their reduced drug metabolizing capacity reserve (11). In contrast to that, Shulman and Ozdemir supposed that cytochrome P450 (CYP) 2D6 activity, which represents the main enzymatic pathway for the antipsychotic agent (12–14), does not change with age (15). Although no enzymatic change was observed, the PK of the active moiety (sum of risperidone and 9-hydroxyrisperidone) potentially vary in the different types of CYP2D6 metabolizers because of age-related changes. In relation to this, a population pharmacokinetic analysis identified age as a significant covariate on the 9-hydroxyrisperidone clearance (16). However, the therapeutic reference range of the active moiety amounts to 20–60 ng/mL published by Hiemke et al. (17). While the most recommended dose in the treatment of schizophrenia in adult patients amounts to 6 mg/day (18), dose reductions should be taken into account for the elderly (19). Therefore, a unifying concept to determine which type of CYP2D6 metabolizer might be more at risk of PK adverse effects would be a benefit due to a greater heterogeneity in the elderly compared to the young adults (11).

Though risperidone metabolism is regulated by the genetic polymorphism of *CYP2D6*, only little is known about correct dosing strategies in the different types of CYP2D6 metabolizers. Based on > 100 allelic variants of the *CYP2D6 *genotype, four different phenotypes have been proposed (20,21): extensive metabolizer (EM), intermediate metabolizer (IM), poor metabolizer (PM), and ultra-rapid metabolizer (UM). To ensure the best therapy for each patient, dose adjustment for genetically caused differences in the plasma concentrations must be considered especially in the elderly to reduce ADR and discontinuations due to ADR in different CYP2D6 phenotypes. So, the aim of the current investigation was to extrapolate a previously published physiologically based pharmacokinetic (PBPK) model to analyze the impact of age-related changes in different types of CYP2D6 metabolizers on the PK of risperidone and 9-hydroxyrisperidone (22). The second aim was to apply calculated risperidone/9-hydroxyrisperidone ratio to classify geriatric patients in different CYP2D6 metabolizers using a PBPK approach. Lastly, we analyzed changes in the PK between the elderly and young adults to finally optimize standard dosage regimes in different CYP2D6 metabolizers. To our knowledge, this is the first study examining the age-related effects of CYP2D6 phenotype-related physiological alterations on the PK of risperidone using a PBPK approach.

## Materials and Methods

### Physiologically Based Pharmacokinetic Modelling

A previously published whole-body PBPK model for risperidone and 9-hydroxyrisperidone was used (22). The modelling was performed using PK-Sim®/MoBi® version 7.3.0 as part of the Open System Pharmacology Suite (23). This package contains a freely available whole-body PBPK simulation software, allowing the prediction of PK, especially for drugs in humans, as well as in several mammalian organisms (23). With the help of PBPK modelling it is possible to run virtual clinical trials of specific subpopulations to overcome sparse clinical data. For a detailed description of the generic model structure, consisting of 17 different compartments, and the input/output parameters, please refer to the user software manual (24), Willmann et al. (25) or Kuepfer et al. (26).

### Model Extrapolation

The published PBPK models were utilized to predict age-related changes in PK values of risperidone and 9-hydroxyrisperidone from young adults (18–35 years) to the elderly aged 65+ years. For model extrapolation the modelling software considered age-dependent anatomical and physiological changes, e.g. weight of the organs and tissues including blood content, blood flow rates, body composition, etc. (27). Age-related changes in CYP2D6 activity were not implemented, as there does not appear to be an age-related decline in the activity of this enzyme in the elderly (15,28). However, significant interactions can occur from concomitant medication, either competitively or noncompetitively inhibiting* CYP2D6* (28). Consequently, no compound-related modifications were conducted for model extrapolation.

### Model Verification

#### Study Data

All PBPK models were verified in the elderly using published clinical data of 20 geriatric inpatients aged 55 years of age or older, admitted to the inpatient programs of the Western Psychiatric Institute and Clinic (3811 O’Hara Street, Pittsburgh, PAP 15213) between November 1996 and March 1998 (29). All patients were identified through daily reviews of pharmacy records, prescribed risperidone and treated under naturalistic conditions. A specification regarding the ethnic origin of the inpatients and the type of CYP2D6 metabolizer was not made.

#### Data Implementation

For model verification, only the elderly inpatients (aged 65+ years) were included into PBPK simulation. Thereby, a total of 17 geriatrics and their corresponding 17 plasma samples were implemented. Calculated creatinine clearance of the subjects were not considered in modelling, as urine collection took place over a period of 8 h. For a correct quantitative analysis, it is necessary to measure the total amount of solutes excreted in a 24-h period, because many solutes exhibits diurnal variations (30). For modelling, all geriatric inpatients were classified as European, as no information of the ethnic origin was provided and the value of age for white Americans must be less than or equal to 81 years, stated by the software (24). Detailed demographic data and patient characteristics are summarized in Table 1.

**Table 1:** Overview of enrolled geriatric inpatients characteristics for PBPK modelling. EM: extensive metabolizer; f: female; IM: intermediate metabolizer; m: male; PM: poor metabolizer; UM: ultra-rapid metabolizer

Patient	Gender	Age (years)	Weight (kg)	Dose (mg/day)	Risperidone concentration (μg/L)	9-hydroxy-risperidone concentration (μg/L)	Risperidone/9-hydroxyrisperidone Ratio	Predicted Phenotype
4	f	69	45.8	1.00	6.56	6.54	1.00	IM
5	f	69	47.2	1.50	0.52	4.38	0.12	UM
6	f	73	76.7	1.00	4.51	5.09	0.89	IM
7	f	73	89.4	0.75	0.60	7.80	0.08	UM
8	f	77	55.3	0.50	1.99	4.01	0.50	IM
9	f	78	62.1	3.00	1.38	5.12	0.27	EM
10	m	79	88.0	1.00	0.32	5.38	0.06	UM
11	m	79	77.6	2.50	1.40	15.8	0.09	UM
12	f	80	59.0	0.50	2.66	4.84	0.55	IM
13	m	80	58.5	1.00	11.2	6.30	1.78	IM
14	f	81	69.9	2.00	2.04	13.0	0.16	UM
15	f	81	49.0	1.50	6.33	1.87	3.39	PM
16	m	82	63.1	1.00	2.03	9.67	0.21	EM
17	f	82	50.8	0.75	0.34	6.96	0.05	UM
18	m	86	69.9	1.00	22.3	24.8	0.90	IM
19	m	88	57.2	1.00	2.40	6.40	0.38	EM
20	f	91	68.0	0.50	3.28	7.92	0.41	EM

#### Phenotyping Using Metabolic Ratio

All geriatric inpatients were individually classified into the different types of CYP2D6 metabolizer, according to calculated risperidone/9-hydroxyrisperidone ratio (also called metabolic ratio), which is a marker for CYP2D6 activity. Here, the increase in the number of active *CYP2D6* alleles indicates a decrease risperidone/9-hydroxyrisperidone ratio. The following mean values were used for phenotyping, taking time-dependency of the ratio into account: 0.16 (UM), 0.27 (EM), 0.72 (IM), and 5.00 (PM) (22). The individual classification of the 17 geriatric inpatients was based on the distance to the respective mean ratio. A detailed overview about metabolic ratio and its predicted phenotype is provided in Table 1.

#### Modelling and Simulation

For model verification, 17 different simulations were built, representing each geriatric inpatient. Here, the individual predicted CYP2D6 phenotype was taken into account. Furthermore, all generated PBPK individuals were in agreement with the corresponding patient characteristics in terms of gender, age and weight (Table 1). Patient-specific daily doses of risperidone (0.5–3 mg/day) were considered in each simulation (Table 1). To ensure a steady-state condition, modelling was carried out over a time frame of 6 days (144 h) and 120 h was set as sampling relevant dosing time point. All risperidone’s and 9-hydroxyrisperidone’s plasma samples of Maxwell et al. were used as observed data in modelling and simulation (Table 1). Maxwell et al. reported that plasma samples were obtained in the morning 9–13 h after the last risperidone dose (in one not defined inpatient 15.5 h). Since no precise patient-individual sampling time points are available, time points (129–135.5 h) were estimated visually by comparing the measured with the predicted plasma concentrations horizontally. A visual comparison was also performed regarding dosing interval (once a day or bi-daily). Simulations were defined as being successful, if the predicted plasma concentration-time profiles of risperidone and 9-hydroxyrisperidone were within the 0.5- to 2-fold interval of the observed concentration of each individual inpatient.

### Prediction of the Pharmacokinetics

After the successful extrapolation and verification of PBPK models, they were utilized to predict age-related changes in different PK values from young adults (18–35 years) to the elderly aged 65+ years. All elderly patients were further subdivided into the following classes: young-olds (65–74 years), medium-olds (75–84 years), and oldest-old (85–100 years) according to the Recommendations on Definition and Classification of Age provided by the National Statistical Standards in November 2016 (31). No adjustment to the PBPK models were done when extrapolating simulations to the different age-classes. Simulations were carried out with the aid of a computer-assisted Monte-Carlo method on populations consisting of 1000 virtual individuals (proportion of women: 0.5) over a time period of 1 week (168 h) including risperidone and 9-hydroxyrisperidone. Steady-state simulations were performed starting with 0.5 mg twice a day for 1 day, resulting in a dose increase of up to 1 mg twice a day (2 mg/day). The different types of CYP2D6 metabolizers (EM, IM, PM, and UM) were considered separately.

### Statistical Analysis

The evaluation of the extrapolated PBPK models was carried out in accordance with the Guideline on the Qualification and Reporting of Physiologically Based Pharmacokinetic Modelling and Simulation (32). Different PK values, such as the median area under the systemic drug concentration-time profile (AUC), maximum plasma concentration of the drug (C_max_), time it takes until the drug reached the C_max_ (t_max_), as well as the terminal half-life time (t_1/2_), were calculated by PK-Sim®.

The quality of PBPK model verification was analyzed using predicted versus observed data plots and weighted residuals versus observed plasma concentrations. To describe model accuracy and precision, prediction error (PE), mean prediction error (MPE) and mean absolute prediction error (MAPE) were calculated according to Eqs. 1–3.

1 $$ \mathrm{PE}\ \left[\%\right]=\frac{predicted- observed}{observed}\ x\ 100 $$

2 $$ \mathrm{MPE}\ \left[\%\right]=\frac{1}{n}\ x\ {\sum \limits}_{i=1}^n PEi $$

3 $$ \mathrm{MAPE}\ \left[\%\right]=\frac{1}{n}\ x\ {\sum \limits}_{i=1}^n\left| PEi\right| $$

All statistical and graphical analytics were performed in R (version 3.4.2; R Foundation for Statistical Computing, Vienna, Austria) (33).

## Results

### CYP2D6 Phenotyping Using Metabolic Ratio

**Fig. 1:**
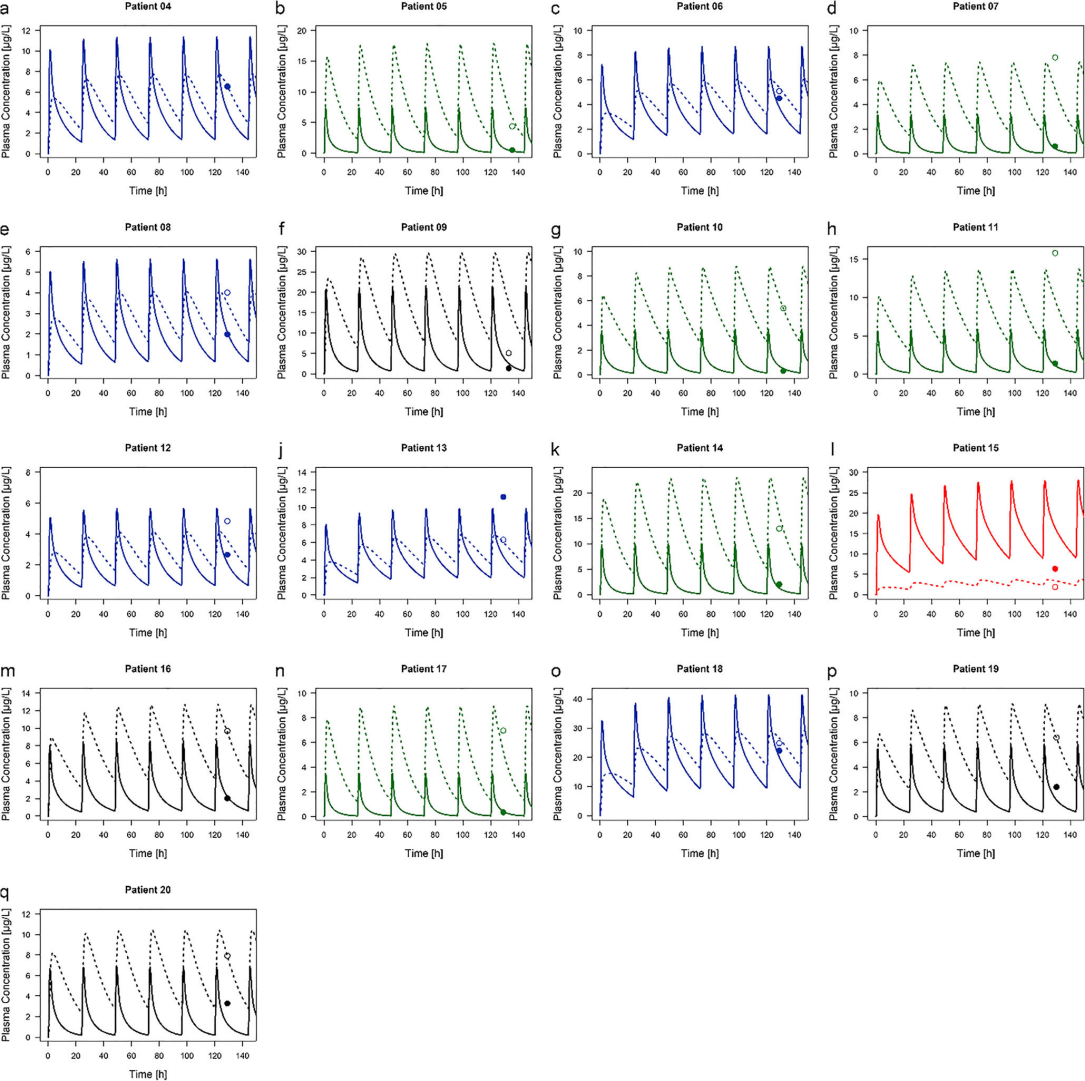
**a**-**q** Plasma concentration- time curve profiles of risperidone and 9-hydroxyrisperidone. Solid lines indicate the mean prediction of risperidone. Dashed lines indicate the prediction of 9-hydroxyrisperidone. Circles indicate observed plasma concentrations. Black colors represent extensive metabolizers, blue colors intermediate metabolizers, red colors poor metabolizers and green colors ultra-rapid metabolizer

Based on a previously developed risperidone/9-hydroxyrisperidone ratio for each CYP2D6 phenotype, the ratio was applied to clinically observed data of 17 geriatric inpatients treated under naturalistic conditions to identify the individual type of CYP2D6 metabolizer (Table 1). Overall, ten geriatric inpatients show a non-reduced metabolic capacity for CYP2D6 (EM and UM) and six inpatients a reduced function (IM) whereas one of the 17 geriatric inpatients (5.88%) was identified as PM, showing a plasma concentration of 6.33 μg/L for the parent drug compared to a value of 1.87 μg/L for the active metabolite. Thus, calculated metabolic ratio (risperidone/9-hydroxyrisperidone) amounts to 3.39 illustrating a higher metabolic ratio compared to the metabolic ratio of the other 16 inpatients (range: 0.05–1.78). An overview of all plasma concentration-time curve profiles is provided in Fig. 1.

### PBPK Model Application in the Elderly

**Fig. 2:**
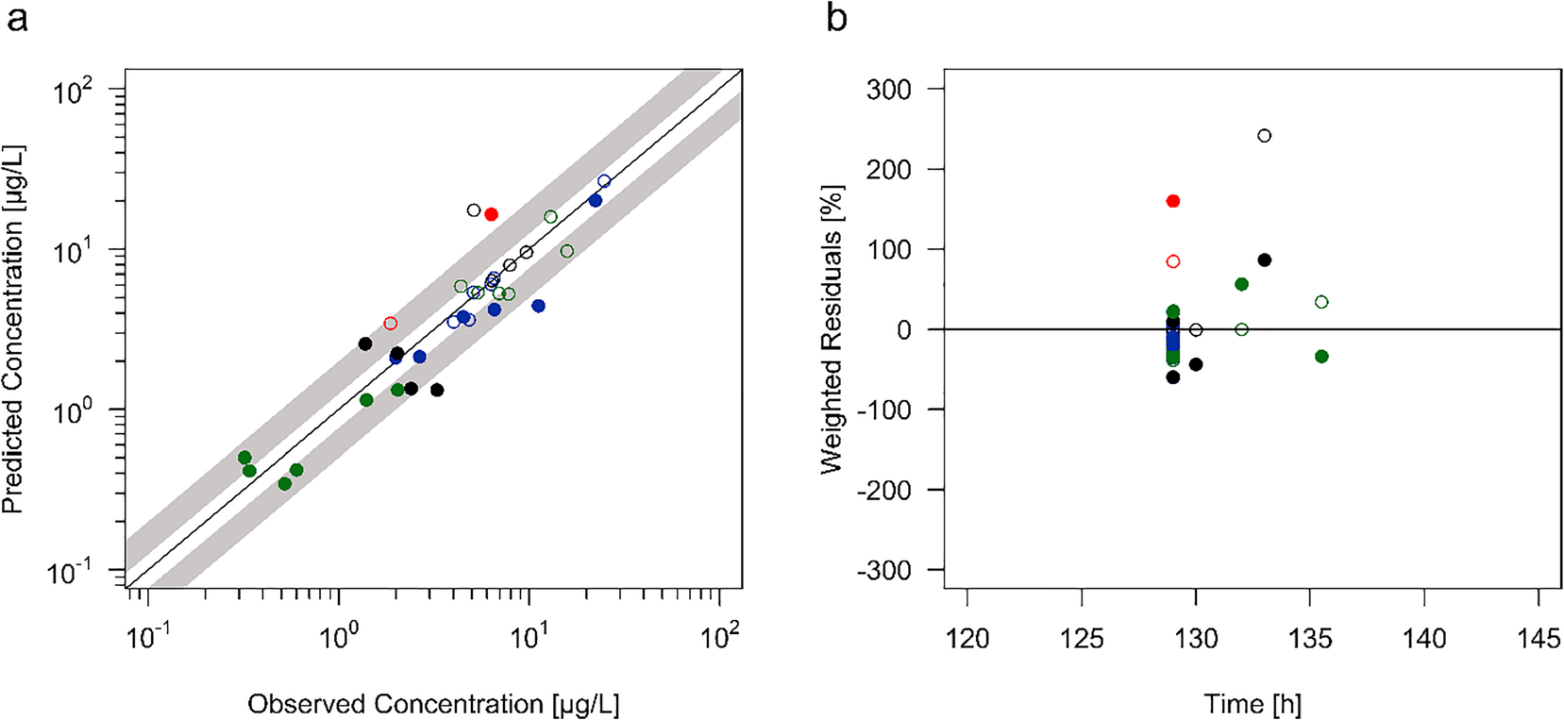
**a** Plot of predicted versus observed data for model prediction of risperidone and 9-hydroxyrisperidone plasma concentrations in all 17 geriatric inpatients. The solid black line indicated the line of identity, filled (risperidone) and open (9-hydroxyrisperidone) circles indicate observed data. Black circles represent extensive metabolizers, blue circles intermediate metabolizers, red circles poor metabolizers and green circles ultra-rapid metabolizers. The white area represents the 1.25-fold error range and the grey area the 2-fold error range. **b** Weighted residuals versus observed risperidone and 9-hydroxyrisperidone concentrations for all 17 geriatric inpatients. The solid black line indicated the line of identity, filled (risperidone) and open (9-hydroxyrisperidone) circles indicate observed data. Black circles represent extensive metabolizers, blue circles intermediate metabolizers, red circles poor metabolizers and green circles ultra-rapid metabolizers

After PBPK model extrapolation to the elderly, the models accurately describe the plasma concentrations of the parent drug and its metabolite during steady-state condition in elderly adults aged ≥ 65 years. Predicted plasma concentrations of risperidone and 9-hydroxyrisperidone were in close agreement to the observed clinical data of each individual, as can be seen by the individual plasma concentration-time curve profile of each geriatric inpatient (Fig. 1). 52.9% of all predicted plasma concentrations were within the 1.25-fold error range and 88.2% were within the 2-fold error range in the plot of predicted versus observed data (Fig. 2). A total of four (three risperidone and one 9-hydroxyrisperidone) plasma concentrations out of 34 samples (11.8%) were outside of the 2-fold error range, indicating a possible influence of individual concomitant medication on the metabolizing enzymes CYP2D6 and CYP3A4. In accordance with this, the visual comparison of the plasma concentration-time curve profile of these inpatients show a deviation between the observed clinical data and the predicted values (Fig. 1). Except from those four geriatric inpatients the developed PBPK models show a high accuracy illustrated by a minimal bias (MPE range: −43.8% to 56.7% for risperidone and − 38.6% to 34.4% for 9-hydroxyrisperidone) and a good precision (MAPE range: 5.7% to 56.7% for risperidone and 0.3% to 38.6% for 9-hydroxyrisperidone). In contrast to that a higher bias and lower precision can be seen in the remaining four geriatric inpatients. Here the MPE amounts to a range of −59.7% to 159.9% for risperidone and − 3.9% to 241.8% for 9-hydroxyrisperidone and the MAPE amounts to a range of 59.7% to 159.9% for risperidone and 0% to 241.8% for 9-hydroxyrisperidone, respectively (Table 2). In accordance with the respective plasma concentration-time profiles of those four geriatric inpatients, the calculated MPE and MAPE suggesting a possible impact on the metabolism of risperidone, resulting in an over-prediction of the PK values especially of the active metabolite. In addition, the plot of predicted versus observed data support these observations (Fig. 2).

**Table 2:** Calculated ratios and mean absolute prediction error (MAPE) for the prediction of plasma concentrations of risperidone and 9-hydroxyrisperidone

Patient	Risperidone		9-hydroxyrisperidone	
	predicted/observed ratio	MAPE	predicted/observed ratio	MAPE
4	0.64	35.9	1.01	0.7
5	0.66	33.7	1.34	34.4
6	0.84	16.1	1.06	5.9
7	0.70	29.8	0.67	32.7
8	1.06	5.7	0.88	12.4
9	1.86	86.1	3.42	241.8
10	1.57	56.7	1.00	0.3
11	0.82	18.3	0.61	38.6
12	0.80	19.9	0.75	25.2
13	0.40	60.3	0.96	3.9
14	0.65	35.2	1.23	22.9
15	2.60	159.9	1.84	84.4
16	1.10	10.3	0.99	1.3
17	1.22	21.9	0.76	23.7
18	0.90	9.6	1.07	7.1
19	0.56	43.8	0.99	0.6
20	0.40	59.7	1.00	0.0

### Age-Related Changes in the Pharmacokinetics

**Fig. 3:**
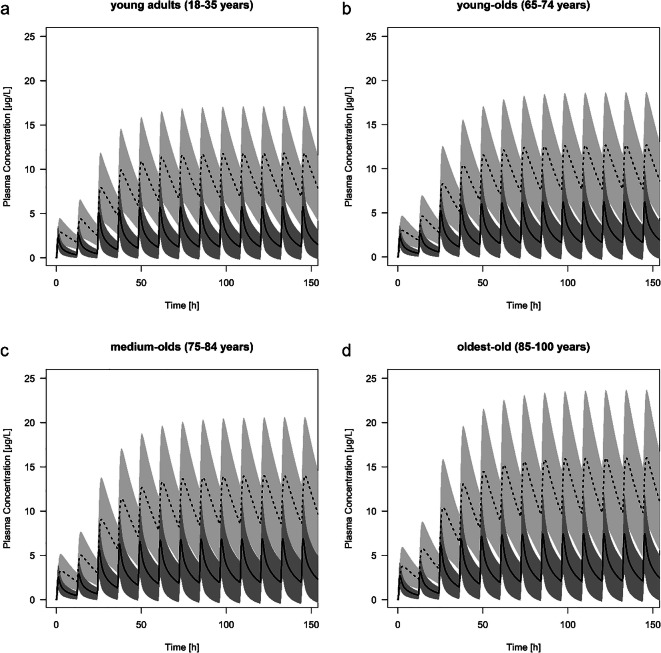
Predicted plasma concentration-time curve profiles (mean and standard deviation) of risperidone (solid line) and 9-hydroxyrisperidone (dashed line) during aging for CYP2D6 extensive metabolizers. Simulations were performed starting with 0.5 mg twice a day for 1 day, resulting in a dose increase up to 1 mg twice a day. **a** young adults (18–35 years). **b** young-olds (65–74 years). **c** medium-olds (75–84 years). **d** oldest-old (85–100 years)

**Fig. 4:**
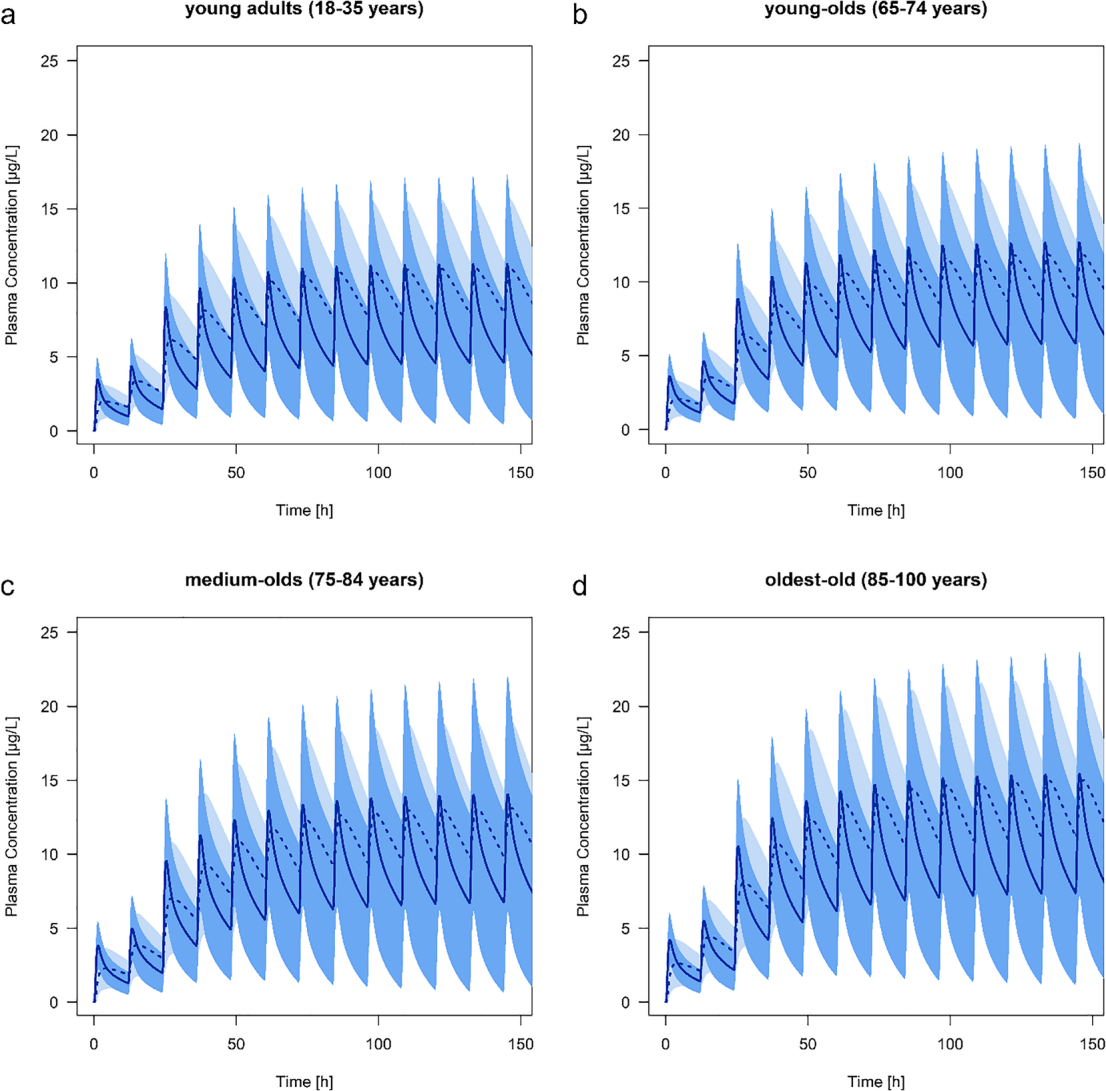
Predicted plasma concentration-time curve profiles (mean and standard deviation) of risperidone (solid line) and 9-hydroxyrisperidone (dashed line) during aging for CYP2D6 intermediate metabolizers. Simulations were performed starting with 0.5 mg twice a day for 1 day, resulting in a dose increase up to 1 mg twice a day. **a** young adults (18–35 years). **b** young-olds (65–74 years). **c** medium-olds (75–84 years). **d** oldest-old (85–100 years)

**Fig. 5:**
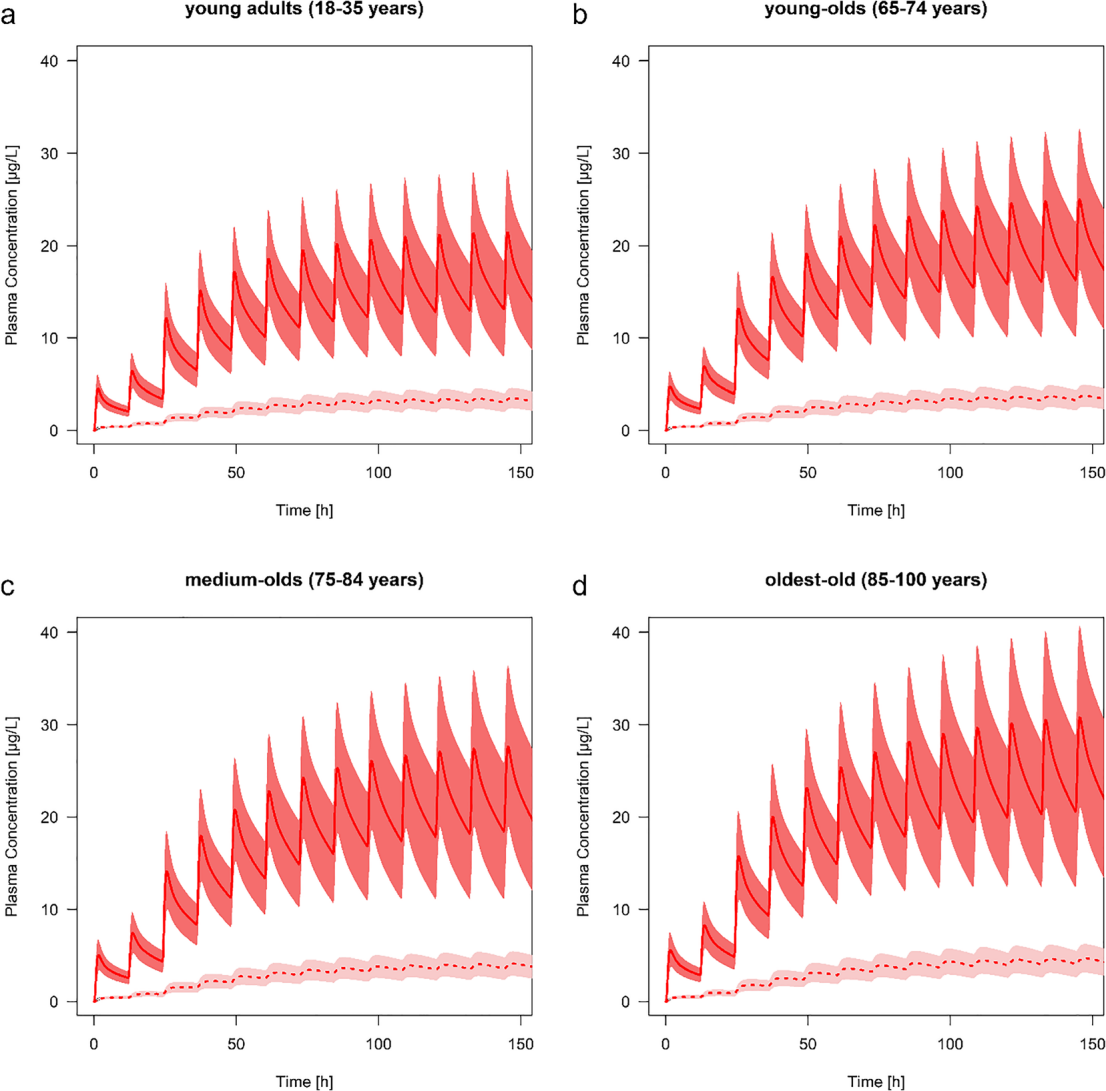
Predicted plasma concentration-time curve profiles (mean and standard deviation) of risperidone (solid line) and 9-hydroxyrisperidone (dashed line) during aging for CYP2D6 poor metabolizers. Simulations were performed starting with 0.5 mg twice a day for one day, resulting in a dose increase up to 1 mg twice a day. **a** young adults (18–35 years). **b** young-olds (65–74 years). **c** medium-olds (75–84 years). **d** oldest-old (85–100 years)

**Fig. 6:**
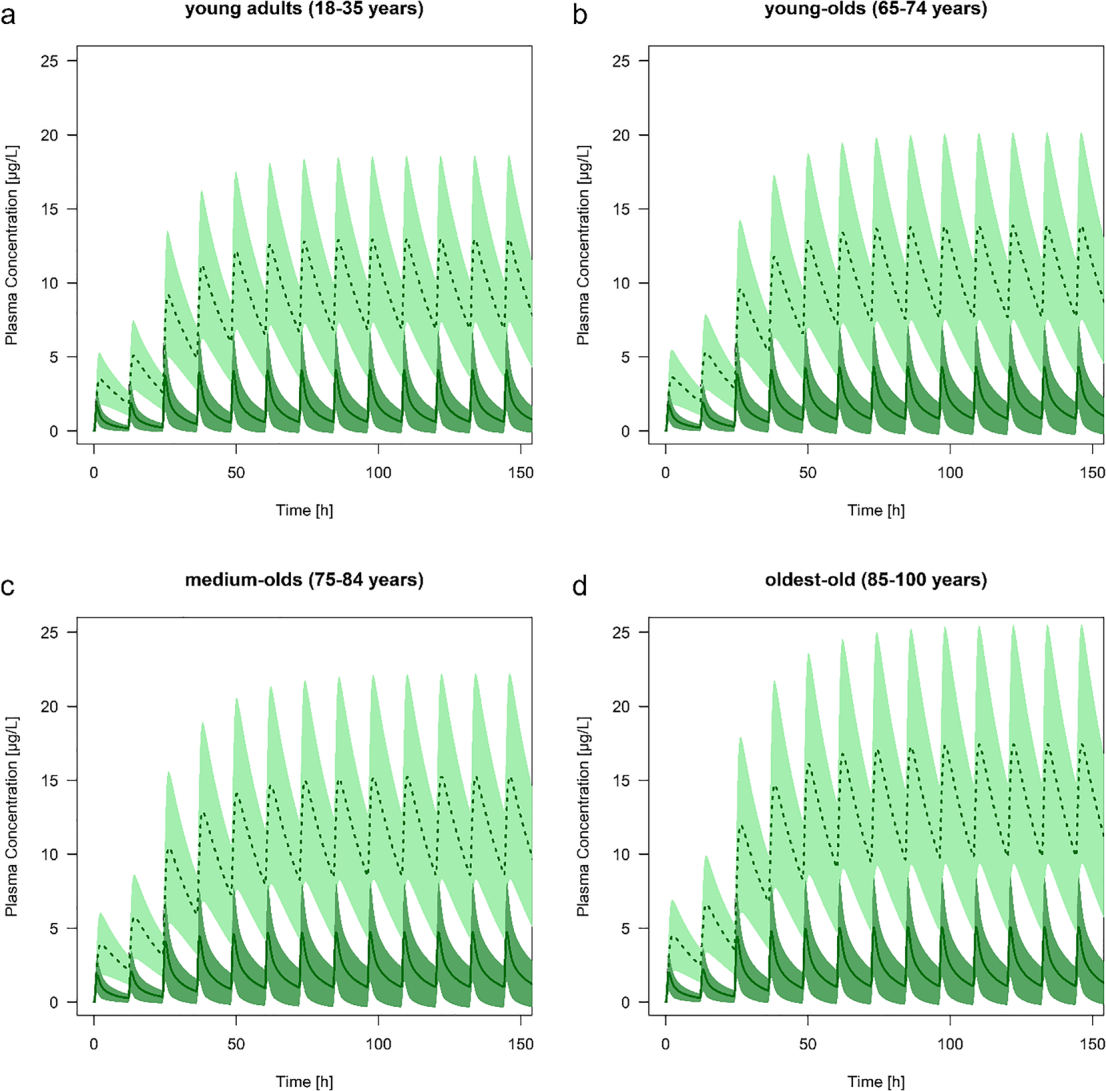
Predicted plasma concentration-time curve profiles (mean and standard deviation) of risperidone (solid line) and 9-hydroxyrisperidone (dashed line) during aging for CYP2D6 ultra-rapid metabolizers. Simulations were performed starting with 0.5 mg twice a day for one day, resulting in a dose increase up to 1 mg twice a day. **a** young adults (18–35 years). **b** young-olds (65–74 years). **c** medium-olds (75–84 years). **d** oldest-old (85–100 years)

The predicted C_max_ of risperidone and 9-hydroxyrisperidone increased with age in all types of CYP2D6 metabolizer. Comparing a population of young adults with oldest-old, the C_max_ during steady-state of risperidone increased with 29% (EM), 37% (IM), 44% (PM) and 24% (UM) and for the active metabolite with 36% (EM), 37% (IM), 35% (PM) and 35% (UM). In practice, the predicted active moiety of EM (1 mg twice a day) will increase by the amount of 17.4 μg/L (young adults) to 23.2 μg/L (oldest-old), leading to an increase of 33.6% during steady-state. These shift in C_max_ values is also seen in the predicted populations of young-olds (7.54% for EM) and the medium-olds (18.7% for EM) compared to the young adults. The predicted AUC of the oldest-old population showed the highest predicted change with up to a 1.52-fold difference (range: 1.47 to 1.52 according to the different phenotypes) for risperidone and up to 1.39-fold difference (range: 1.34 to 1.39) for 9-hydroxyrisperidone compared to the young adults (Table 3). Overall, the AUC increased with age in all types of CYP2D6 metabolizers (Table 3). In contrast to the C_max_ and AUC, the PK parameter t_max_ did not change with advanced age, neither for the parent drug, nor for the active metabolite (Table 3). The terminal half -life time showed predominantly an increase for the parent drug while aging, compared to the prediction of the medium-old population and the oldest-old to the young adults. Here, CYP2D6 PM showed the highest change with up to a 1.10-fold difference resulting in a predicted t_1/2_ value of 19.4 h for the drug. For 9-hydroxyrisperidone t_1/2_ was independent of age. Plasma concentration-time curve profiles of all types of CYP2D6 metabolizers are provided in Figs. 3, 4, 5 and 6.

**Table 3:** Overview of different pharmacokinetics values for risperidone and 9-hydroxyrisperidone between different age groups: young adults (18–35 years), young-olds (65–74 years), medium-olds (75–84 years), and oldest-old (85–100 years). EM: extensive metabolizer; IM: intermediate metabolizer; PM: poor metabolizer; UM: ultra-rapid metabolizer

		Risperidone			9-hydroxyrisperidone		
		Ratio young-old/young adults	Ratio medium-old/young adults	Ratio oldest-old/young adults	Ratio young-old/young adults	Ratio medium-old/young adults	Ratio oldest-old/young adults
AUC (μg*h/l)	EM	1.20	1.38	1.49	1.09	1.22	1.39
	IM	1.20	1.37	1.51	1.09	1.21	1.38
	PM	1.21	1.36	1.52	1.15	1.18	1.34
	UM	1.19	1.38	1.47	1.09	1.21	1.39
C_max_ (μg/L)	EM	1.08	1.19	1.29	1.07	1.18	1.36
	IM	1.12	1.24	1.37	1.08	1.19	1.37
	PM	1.17	1.29	1.44	1.07	1.18	1.35
	UM	1.06	1.16	1.24	1.07	1.18	1.35
t_max_ (h)	EM	1.00	1.00	1.00	1.00	1.00	1.00
	IM	1.00	1.00	1.00	1.00	1.00	1.00
	PM	1.00	1.00	1.00	1.00	1.00	1.00
	UM	1.09	1.09	1.09	1.00	1.00	1.00
t_1/2_ (h)	EM	1,09	1,15	1,14	1,04	1,04	1,03
	IM	1,07	1,11	1,11	1,04	1,02	1,00
	PM	1,08	1,10	1,10	0,98	0,85	0,74
	UM	1,10	1,17	1,16	1,04	1,04	1,04

## Discussion

In the present study, we analyzed the influence of age-related as well as CYP2D6- and CYP3A4- related physiological changes on the PK of risperidone and 9-hydroxyrisperidone using a PBPK approach. The application of calculated metabolic ratio and changes of PK values for dosage calculation in the elderly were the focus of the investigations.

### PBPK-Based Phenotyping Using Metabolic Ratio

In our study, calculated risperidone/9-hydroxyrisperidone ratio were observed to readily predict CYP2D6 phenotypes of geriatric inpatients using a PBPK approach. Here, modelling based phenotyping was successful in 13 out of 17 geriatric inpatient using metabolic ratio (Fig. 1). The remaining four inpatients showed higher deviations between the observed and predicted plasma concentrations. There are manifold reasons for this. Firstly, all geriatric inpatients enrolled in the published investigation were treated under naturalistic conditions (29). This means, potentially concomitant medication with CYP inhibitors or inducers should be considered, since patients over the age of 65 years often suffer from several diseases. In general, it is known that a CYP2D6 PM is associated with an increase in moderate-to-marked ADR and increased discontinuation due to ADR (34). A metabolic ratio of 3.39, as calculated in one out of the four geriatric inpatients (patient no. 15), indicate a potential CYP2D6 PM phenotype or the influence of a powerful CYP2D6 inhibitor (such as fluoxetine, paroxetine, or bupropion) (34). It is known that patients on strong inhibitors, the enzyme activity score of CYP2D6 is adjusted to 0 and the predicted phenotype is that of a PM (35). In addition, weak or moderate inhibitors leading to a multiplication of the activity score by 0.5 (36,37). Next to the genetic aspects, it is secondly necessary to integrate individual covariates such as illness, especially renal and hepatic impairment into CYP genotyping process. Here, the creatinine clearance of two out of the four geriatric inpatients (patient no. 9 and 15) amounts to 25.0 mL/min/1.73m^2^ and 27.5 mL/min/1.73m^2^ using 8 h urine collection according to Maxwell et al. (29). Apart from an inadequate measurement, both values indicate a severe decrease in glomerular filtration rate (38).

Overall, the new approach demonstrates, that it is possible to determine CYP2D6 phenotypes in the elderly, only with the aid of measured plasma concentrations in combination with the extrapolated PBPK models. This new CYP2D6 phenotyping approach is particularly accomplishable during therapeutic drug monitoring (TDM). A TDM of risperidone and its active metabolite is recommended by many experts because schizophrenia patients often fail to take medication, deviate from the prescribed regimen, or discontinue therapy prematurely (17,39). This also occurs increasingly in the elderly, in whom we could identify age-related changes in the PK of risperidone. The combination of TDM and CYP2D6 phenotyping may be moving to clinical practice to improve safety (ADR) and efficacy (non-response) of pharmacotherapy of risperidone (22,40). Moreover, pharmacogenetic testing, such as the use of metabolic ratio in combination with PBPK modelling, will improve the sensitivity and specificity of conventional drug monitoring by identifying patients showing conspicuous plasma concentrations. The advantage of that PBPK-based phenotyping approach over the standardized CYP genotyping is, that only plasma concentrations of the drug and its active metabolite are needed and no costly CYP genotyping has to be organized. The simple and very quick calculation of the metabolic ratio can be conducted easily by a clinician or other healthcare personnel. Long latency for laboratory results are no longer necessary. In addition, this method determines the phenotype of every schizophrenia patient and not only in patients suspected of altered CYP2D6 gene expression, as done for CYP genotyping. This leads to the fact that PBPK-based phenotyping using metabolic ratio also includes patients for whom a change in the CYP2D6 gene expression was accidentally identified. Even subpopulations like the elderly would benefit from that application. Altogether, when plasma concentrations of risperidone and 9-hydroxyrisperidone are available, e.g. during TDM, the new approach could be used to identify potential CYP2D6 UM, IM or PM and predict their individual dosage regimen with the help of PBPK modelling. This would be particularly important in the elderly (> 65 years) due to age-related changes in the PK of risperidone.

### Pharmacokinetic Changes in the Elderly

The results of the present study show that steady-state plasma concentrations of risperidone and 9-hydroxyrisperidone including the active moiety increase progressively during aging (Figs. 3, 4, 5 and 6). Since CYP2D6 activity does not change with age, higher plasma concentrations in the elderly may be due to physiological changes, like a decrease in renal and hepatic function (15). This stems in part from findings of a general accumulation of antidepressant drug’s active hydroxylated metabolites (e.g. nortriptyline and bupropion) in the elderly supposed by Pollock and colleagues (28). However, human in vitro and in vivo investigations do not uniformly support an age-related decline in hepatic drug metabolism (41–43). It has been found that the PK of the two drugs nortriptyline and desipramine in the elderly is similar to those in younger patients (44). Apart from that, our findings are basically in line with the previous study (28,45). The explanation for an accumulation of 9-hydroxyrisperidone after oral administration of risperidone may be the age-dependent decline in kidney function, leading to an increase in the exposure of the overall active moiety (C_max_ of young adults versus oldest-old +33.6% for EM). As a consequence, geriatric patients might be at increased risk of side effects when exposed to orally administrated risperidone. Based on the predicted higher plasma concentrations in all types of CYP2D6 metabolizers, a reduced daily dosing should be considered in the elderly. In clinical practice, it is therefore important to be aware that daily oral dosing of risperidone generally needs to be lowered by more than 50% to achieve reduced treatment intensity in the oldest patients (3). These observations are in accordance with our developed PBPK models of the different age groups (Fig. 3, 4, 5 and 6). It is recognizable, that dose adjustment for the elderly compared to young adults should be conducted. Here, an accurate TDM, especially for geriatric inpatients showing a metabolic ratio > 1, is highly recommended due to higher plasma concentrations, leading to a higher risk of ADR (Fig. 5) (40). For those patients it is appropriate to identify a potential CYP2D6 PM status via genotyping to avoid supratherapeutic plasma concentrations. Besides higher plasma concentrations, patients above 65 years show a longer elimination t_1/2_ of risperidone compared to young adults. For PM the PK parameter t_1/2_ amounts to 19.4 h for risperidone, which is consistent with previously published studies (t_1/2_ of risperidone: 20 h) (46). In accordance with the prolonged elimination half-life, Snoeck et al. supposed an enlarged AUC in the elderly compared to those of young adults for risperidone as well as for 9-hydroxyrisperidone (46). The AUC was predicted to increase progressively with age in all types of metabolizers for both, the drug and its metabolite (Table 3), which can mainly explained by a diminished creatinine clearance and decreased renal clearance during aging (46).Overall, the new investigations illustrate on the one hand age-related changes in the PK of the drug and its active metabolite. One the other hand the PK-based PBPK models also showed that an age-appropriate dosing of risperidone is essential for a successful therapy. Thus, in patients over the age of 65 years, dose reduction for all types of metabolizers should be considered based on the current findings in order to avoid supratherapeutic plasma concentrations.

### Impact of Age on the Pharmacodynamics

Finally, it should be mentioned that age-related changes in the physiology and biology of the elderly also affect the pharmacodynamics (PD) of drugs. Although risperidone has a linear concentration side-effect profile in the elderly, neuronal changes such as a decrease in dopamine or acetylcholine can lead to greater sensitivity of dopamine receptor D2 antagonists and antimuscarinic agents (47). Thus, the predicted higher plasma concentrations of risperidone and 9-hydroxyrisperidone may lead to a more relevant impact on the PD of the elderly compared to young adults. In contrast to that, the impact of different CYP2D6 metabolizers on the PD is not clear.

### Limitation

A clear limitation of our study is that clinical PK data for genotyped individuals aged older than 65 years are sparse and thus, the conducted prediction and simulation results need to be interpreted with caution. For the observed data used in our study, no prior CYP genotyping was performed, so phenotyping was only based on calculated metabolic ratio. In addition, several individual data were not provided regarding potential liver cirrhosis (48), renal impairment (49), heart disease (50) or concomitant medication (51) of each geriatric patient, which would be helpful for modelling. It is known that those patient related circumstances can change physiology, resulting in a variation of individual PK. Finally, a PK/PD model especially for patients above the age of 65 years would be helpful to understand the impact of age-related changes, resulting in an optimization of the individualised treatment strategy in the elderly according to CYP2D6 phenotype.

## Conclusion

The present study generated and verified a PBPK model for risperidone and 9-hydroxyrisperidone for different CYP2D6 metabolizers over the age of 65 years. After oral administration of risperidone, a progressive increase in the predicted plasma concentrations of risperidone and its active metabolite 9-hydroxyrisperidone was observed while aging. Although the prescribed dosage is generally lower in geriatric patients, a close TDM of both risperidone and 9-hydroxyrisperidone is highly recommended for all types of CYP2D6 metabolizers. As part of the TDM, CYP2D6 phenotyping by calculating the metabolic ratio in combination with PBPK modelling should be considered, on the one hand to identify patients who show changes in their CYP2D6 gene expression, and on the other hand to predict the individual dosing regimen. Overall, using this new approach, the identification of potential CYP2D6 PM, IM, or UM is cheaper, faster and easier compared to conventional CYP genotyping and can be used to improve patient’s safety and ensure the best therapy for each patient over the age of 65 years.

### ACKNOWLEDGMENTS AND DISCLOSURES

Lisa Alina Kneller and Georg Hempel have no potential conflicts of interest to declare.

## Abbreviations

ADR: Adverse drug reactions
AUC: Area under the systemic drug concentration-time profile
C_max_: Maximum plasma concentration of the drug
CYP: Cytochrome P450
EM: Extensive metabolizer
IM: Intermediate metabolizer
MAPE: Mean absolute prediction error
MPE: Mean prediction error
PBPK: Physiologically based pharmacokinetic
PD: Pharmacodynamic
PE: Prediction error
PK: Pharmacokinetic
PM: Poor metabolizer
TDM: Therapeutic drug monitoring
t_max_: Time it takes until the drug reached C_max_
t_1/2_: Half-life time
UM: Ultra-rapid metabolizer
